# In vitro evaluation of double carbapenem and colistin combinations against OXA-48, NDM carbapenemase-producing colistin-resistant *Klebsiella pneumoniae* strains

**DOI:** 10.1186/s13756-020-00727-4

**Published:** 2020-05-19

**Authors:** Fatma Erdem, Ayham Abulaila, Zerrin Aktas, Oral Oncul

**Affiliations:** 1 Department of Medical Microbiology, Adana City Trainning and Research Hospital, Dr. Mithat Ozsan Boulevard. 4522-1 Yuregir/Adana, Adana, Turkey; 2 Department of Medical Microbiology, Istanbul University Istanbul Medical Faculty, Istanbul, Turkey; 3 Department of Infection Disease and Clinical Microbiology, Istanbul University Istanbul Medical Faculty, Istanbul, Turkey

**Keywords:** Colistin-resistant *Klebsiella pneumoniae*, Time-kill assay, OXA-181, OXA-48, NDM, Whole genome analysis, PCR-based replicon typing, PFGE, MLST

## Abstract

**Background:**

Treatment of pandrug-resistant isolates often necessitates combination therapy. Checkerboard synergy and time-killing assay tests were performed to evaluate the benefits of a triple combination with meropenem, ertapenem, and colistin against 10 colistin-resistant *K. pneumoniae* clinical isolates harboring different β-lactamases. (bla_OXA-48_, bla_NDM_).

**Materials and methods:**

In this study, ertapenem and meropenem (ERT/MEM), meropenem and colistin (MEM/COL), ertapenem, meropenem and colistin (ERT/MEM/COL) combinations were tested using checkerboard techniques and time-kill assays of each antibiotic alone and in combination against 10 colistin-resistant clinical *K. pneumoniae* isolates. An analysis of *K. pneumoniae* isolate B6 using a scanning electron microscope revealed morphologic changes in the cell surface after treatment with each antibiotic both alone and in combination. The whole genome of *K. pneumoniae* KPNB1 was sequenced using an Ion Torrent PGM sequencer.

**Results:**

According to the checkboard results, synergistic combinations were observed with ertapenem/meropenem (5/10 isolates), meropenem/colistin (7/10) and ertapenem/meropenem/colistin (9/10); no antagonism was observed for all combinations. For the time-kill assay results; synergism and bactericidal effects were observed with meropenem/colistin (10/10) and with ertapenem/meropenem/colistin (10/10) combinations, and an indifference effect was observed with the ertapenem and meropenem (10/10) combination. Strain number 1 was found 100% identical to *Klebsiella pneumoniae subsp. pneumoniae* HS11286 according to the outcomes of complete genome sequence analysis, and the strain carried the genes bla_OXA-181_, bla_CTXM-15_, blaNDM, *arr-3, aac (6′)-Ib-cr, rmtF*, and *catB1.*

**Conclusion:**

Using double carbapenem antibiotics with colistin could be a potential alternative to treat colistin and carbapenem-resistant *K. pneumoniae*. The present study is the first Turkish report of OXA–181-type carbapenemase causing colistin resistance.

## Introduction

Infections due to multiple drug-resistant (MDR) Gram-negative bacteria have seriously increased worldwide*.* Carbapenems and colistin have been the treatment of choice for serious infections due to MDR gram negative rods but unfortunately resistance to carbapenems and colistin compromise the treatment options [1]. *K. pneumoniae* strains show high levels of resistance to carbapenems and other antimicrobial classes, with increasing reports of colistin resistance [2, 3]. Colistin resistance in *Klebsiella pneumoniae* is related to modifications of lipopolysaccharide (LPS) by the addition of cationic charges, which decreases the affinity of polymyxins to the LPS target. The modification of LPS is mediated by the pmrHFIJKLM operon, regulated by the PhoPQ and PmrAB two-component systems. A small transmembrane protein MgrB negatively regulates the PhoPQ system by interaction with the sensor kinase PhoQ in the periplasmic domain, preventing activation of the pmrHFIJKLM operon [4, 5]. Other limitations of colistin treatment are toxicity and adverse effects [6]. The toxicity of colistin is considered to be dose-dependent and dose can be decreased by using colistin in a combination [7].

Ceftazidime/avibactam or ceftolozane/tazobactam like regimens are the other treatment options for the pandrug-resistant *K.pneumoniae* strains. Avibactam is a synthetic non-β-lactam β-lactamase inhibitor that inhibits the activities of Ambler class A and C- β lactamases and some Ambler class D enzymes, including bla_KPC_ carbapenemases, AmpC, and OXA-48-like carbapenemases, respectively. However, it does not inhibit metallo-β-lactamases, such as bla_VIM_ or bla_NDM_ variants [8]. Otherwise, avibactam protects aztreonam from hydrolysis by β-lactamases. So, when aztreonam is combined with avibactam, a synergistic effect occurs against bla_NDM_-producing *Enterobacteriaceae* [9]*.* On the other hand, ceftazidime avibactam was combined with either amikacin or meropenem against four KPC-producing *K. pneumoniae*. Synergistic effects were observed in vitro in time-kill assays and increased survival rates were observed in an in vivo model with these combinations [10].

Combinations of different antimicrobials against MDR *K. pneumoniae* isolates were investigated by others. Elamam et al. tested two-drug combinations of antimicrobials against 12 polymyxin B-resistant *K. pneumoniae* isolates. Synergistic effects were observed with polymyxin B- rifampin, doxycycline-polymyxin B, and tigecycline-polymyxin B, but no interaction was observed with a polymyxin B-gentamicin combination [11, 12]. Furthermore, in another study, a synergistic effect was determined with ertapenem and meropenem combinations against both bla_KPC_ and bla_OXA-48_ producers, but not with bla_NDM_ producers [13]. Combination with imipenem is an alternative option. However, poor activity was observed with imipenem against bla_OXA-48_-producing isolates in invivo experiments [14, 15].

New therapeutic approaches are needed because pandrug-resistant *Klebsiella pneumoniae* strains have been increasingly reported worldwide. Combination antibiotic therapy is an efficient approach to treating these infections. Synergy tests might be useful in selecting the best antimicrobial combination for infections due to MDR and PDR *Klebsiella pneumoniae* [16]. We aimed to evaluate the activities of ertapenem and meropenem (ERT/MEM), meropenem and colistin (MEM/COL), ertapenem, meropenem and colistin (ERT/MEM/COL) combinations against 10 colistin-resistant *K. pneumoniae* clinical isolates harboring different β-lactamases (bla_OXA-48_, bla_NDM)_ [14]).

## Materials and methods

### Strain collection and antimicrobial susceptibility tests

A collection of 10 MDR and bla_OXA-48_ and/or bla_NDM_ carbapenemase-producing *K. pneumoniae* clinical isolates were studied because a few MDR *K. pneumoniae* strains have been isolated in our hospital since 2016. The isolates were obtained from clinical samples of hospitalized patients. Antimicrobial susceptibility and MIC characterization was performed using gradient tests. For ertapenem, meropenem, imipenem and colistin, MICs were determined using broth microdilution method. Susceptibility results were interpreted according to the Clinical Laboratory Standards Institute (CLSI) clinical breakpoint guidelines [17]. Beta lactamase activity of isolates was investigated using the disk diffusion test, E test, Blue-Carba (BCT), and Modified Hodge test (MHT).

### Synergy tests

Ertapenem/meropenem (ERT/MEM), meropenem/colistin (MEM/COL) and ertapenem/meropenem/colistin (ERT/MEM/COL) combinations were tested using the checkerboard technique and time-kill assays, with each antibiotic alone and in combinations.

#### Checkerboard technique

The overnight cultures (initial inoculum of 10^5^–10^6^ CFU/mL) were performed with ERT, MEM, and COL alone as well as in combinations. For each strain and antibiotic, the selected concentration ranges were based on the (minimum inhibitory concentrations) MICs of antibiotics. Five increasing (4-fold) concentrations (0.125× MIC to 2× MIC) were used, Interpretation of the checkerboard results was based on the following: fractional inhibitory concentration (FIC) values of ≤0.5 indicate synergy, FIC values of 0.5 to 4 indicate no interaction, and FIC values of > 4 indicate antagonism. The reduction of the original inoculum by ≥3log_10_ CFU/mL was considered bactericidal and a reduction of ≥2log_10_ CFU/mL by the antibiotic combination compared with that of the most active compound was defined as synergism. Indifference was defined as a ≤ 1 log_10_ reduction with the combination compared with that obtained with the most active single agent [18].

#### Time-kill assays

Overnight cultures (initial inoculum of 10^5^–10^6^ CFU/mL) were performed with ERT, MEM, and COL alone as well as in combinations. ERT, MEM, and COL corresponding Cmax serum concentrations (μg/mL) 150, 40, and 10, respectively, were used in all experiments. In vitro activity was assessed at 1, 2, 4, 6, 8, and 24 h. The effect of colistin and its association were also assessed at 30 min [19].

### Scanning electron microscope (SEM)

Morphologic changes; An analysis of *K. pneumoniae* isolate B6 using an SEM revealed morphologic changes on the cell surface after treatment with each antibiotic both alone and in combination.

Overnight cultures (initial inoculum of10^5^–10^6^ CFU/mL) were performed with ERT, MEM, and COL alone as well as in combinations. ERT, MEM and COL corresponding Cmax serum concentrations 150, 40 and 10 μg/mL, respectively, were used and in vitro activity was assessed at 1 h. The tubes were incubated at 37 °C in a shaking water bath for 1 h and then centrifuged at 3220 g for 10 min. The bacterial cells were fixed with 2.5% glutaraldehyde before being washed and resuspended three times in PBS. The bacterial cultures were incubated on polyethylenimine-coated coverslips (22 mm × 22 mm) for 1 h and immersed for a further hour in 2.5% glutaraldehyde in PBS before rinsing in PBS for 10 min, three times. Dehydration was then performed using increasing concentrations of ethanol in water (10, 30, 50, 70, 90 and 100%) for 10 min in each step. The coverslips were air-dried prior to mounting on 25-mm aluminum stubs with double-sided carbon tabs. Silver liquid was applied to the edges of each coverslip, and these were then dried and gold coated in an SC7620 sputter coater (QUORUM TECHNOLOGIES, Ashford Kent, UK). The cells were imaged by using a Quanta FEG 450 SEM (FEI, Hillsboro, OR, USA) [20].

### PFGE (pulse-field gel electrophoresis) and MLST (multi-locus sequence typing)

Genomic DNA was prepared in agarose blocks and digested with the restriction enzyme XbaI. The DNA fragments were separated for 20 h at 6 V/cm and 14 °C with initial and final pulse times of 0.5 and 30 s, respectively [21]. Multi-locus sequence typing (MLST) of *K. pneumoniae* was performed as described by Diancourt et al. [22]. DNA sequences were uploaded into the MLST database (http://bigsdb.web.pasteur.fr/klebsiella/klebsiella.html) and allelic numbers and sequence types (STs) were obtained.

### Genotypic detection of resistance genes

PCR (Polymerase Chain Reaction) and sequencing was used to screen for the presence of genes encoding for colistin (pmrA, *pmrB, phoP, phoQ, and mgrB*); Class A (bla_KPC_, bla_GES_), Class D (bla_OXA-48_,bla_OXA-181_) and Class B (bla_NDM_, bla_IMP_, bla_VIM_, bla_SPM_, bla_SIM_) carbapenemases and ESBL (bla_CTX-M_, bla_TEM_, bla_SHV_) [23, 24]. In addition, plasmid-borne 16S rRNA methylases, including *armA, rmt* (A-E), *npmA*; plasmid-borne quinolone genes (*qnrA, qnrB, qnrS, qepA, aac (6′)-Ib-cr*) and AmpC beta lactamases genes (*DHA, ACC, FOX, MOX, CIT AND EBC*) were screened [24–27]. The results were confirmed by sequencing.

### Whole-genome sequencing (WGS)

In order to determine the genetic basis of pandrug-resistance, we interrogated the genome to identify acquired and intrinsic resistance genes. Chromosomal and plasmid genomes were sequenced using an Ion Torrent PGM sequencer (Thermo Scientific, Bremen, Germany), with 316 v2 chip sequencing, generating 100 base-paired end reads [28].

### Plasmid typing

Plasmid typing analysis was performed using multiplex PCR-based replicon typing (HI1, HI2, I1, I2, X1, X2, L/M, N, FIA, FIB, FIC, FII, FIIS, FIIK, W, Y, P, A/C, T, K, U, R, B/O, HIB-M), as described by Caratolli et al. [29].

## Results

All strains were resistant to all tested antibiotics except tigecycline and amikacin (5/10), and all NDM-1–producer isolates were resistant to amikacin. They were resistant to doripenem (MICs ranging from 4 to 32 mg/L), ertapenem (64 to > 128 mg/L), and meropenem (16 to 128 mg/L), colistin (4 to 64 mg/L) (Tables 1 and 2).

**Table 1:** MICs (mg/L) of antibiotics against *K pneumoniae strains*

	TGC	OF	AMC	LEV	DOR	PTc	AK	CIP	CRO	CXM	TZ/TZL	IP/IPL	TOB	CT/CTL
**1**	3	> 32	128	> 32	> 32	> 256	> 256	16	> 32	> 256	> 32/> 4	< 4/< 1	192	> 16/> 1
**2**	0.38	> 32	> 32	> 32	> 32	> 256	4	> 32	> 32	> 256	> 32/> 4	< 4/1.5	24	> 16/> 1
**14**	0.50	> 32	> 256	> 32	4	> 256	12	> 32	> 256	> 256	> 32/> 4	4/0.5	24	> 16/> 1
**21**	05	> 32	> 256	> 32	4	> 256	8	> 32	> 32	> 256	> 32/> 4	< 4/1.5	24	> 16/> 1
**22**	1	> 32	> 256	32	> 32	> 256	> 256	> 32	> 32	> 256	> 32/> 4	< 4/< 1	> 1024	> 16/> 1
**24**	0.50	> 32	> 256	32	> 32	> 256	6	> 32	> 32	> 256	> 32/> 4	4/1.5	24	> 16/> 1
**28**	0.38	> 32	> 256	> 32	4	> 256	4	> 32	> 32	> 256	> 32/> 4	< 4/1	32	> 16/> 1
**31**	2	32	> 256	> 32	6	> 256	8	> 32	> 32	> 256	> 32/> 4	< 4/2	32	> 16/> 1
**37**	0.50	> 32	> 256	> 32	4	> 256	> 256	> 32	> 32	> 256	> 32/> 4	< 4/< 1	> 1024	> 16/> 1
**B6**	0.38	> 32	> 256	> 32	12	> 256	> 256	> 32	> 32	> 256	> 32/> 4	< 4/< 1	192	> 16/> 1

*TGC* Tigecycline, *OF* Ofloxacin, *AMC* Amoksicilin clavulonik acid, *LEV* Levofloxacin, *CIP* Ciprofloxacin, *CRO* Ceftriaksone, *CXM* Cefuroxime, *TZ/TZL* Tikarcilin/tikarcilin clavulonic acid, *IP/IPL* Imipenem/Imipenem EDTA, *TOB* Tobramycin, *CT/CTL* Cefotaxime/ cefotaxime clavulonic acid

**Table 2:** Results of synergy tests PCR, REP-PCR, PFGE and MLST for *K. pneumoniae* isolates (*n* = 10)

				Results of Checkerboard Test			Results of Time Kill Assay			MIC (mg/L)
Strain NO	PFGE	GENE	REP-PCR	Ert + mem	Mem + col	Ert + mem + col	Ert + mem	Mem + col	Ert + mem + col	ERT/MEM/COL
**KP1**	ST14	NDM-1 CTX-M-15 TEM, SHV, rmtB, ArmA	INCR, INCH1b, M, INCF1b, INC FII	0.5 0.62 SYN-ADT	0.5 0.75 SYN-ADT	0.28 0.56 SYN-ADT	IND	SYN	SYN	> 128/64/16
**KP2**	ST101	OXA-48 CTX-M-15 TEM, SHV rmtD	INCR, INCL	1–1.5 IND-IND	0.18 0.56 SYN-ADT	0.14 1.06 SYN-IND	IND	SYN	SYN	> 128/32/16
**KP14**	ST101	OXA-48 CTX-M-15 TEM, SHV rmtD	INCR, INCL	0.31 0.56 SYN-ADT	0.25 1.03 SYN-IND	0.14 1.06 SYN-IND	IND	SYN	SYN	32/32/4
**KP21**	ST101	OXA-48 CTX-M-15, TEM, SHV rmtD	INCR	0.5 0.75 SYN-ADT	0.5 0.56 ADT-SYN	0.5 0.62 SYN-ADT	IND	SYN	SYN	128/32/16
**KP22**	ST395	NDM-1,OXA-48 CTX-M-15, rmtC	INCR, INCL	0.5 0.62 SYN-ADT	0.05 0.5 SYN-SYN	0.15 0.53 SYN-ADT	IND	SYN	SYN	128/32/4
**KP24**	ST101	OXA-48 CTX-M-15 TEM, SHV rmtD	INCR, INCL	0.62 0.75 ADT-ADT	0.75 1 ADT-IND	0.13 0.5 SYN-SYN	IND	SYN	SYN	> 128/32/16
**KP28**	ST101	OXA-48, CTX-M-15 TEM, SHV rmtD	INCR, INCL	0.62 0.75 ADT-ADT	0.5 0.62 SYN-ADT	0.75 1 ADT-IND	IND	SYN	SYN	> 128/16/16
**KP31**	ST101	OXA-48 CTX-M-15 TEM, SHV rmtD	INCR, INCL	0.50 1.12 SYN-IND	0.25 0.5 SYN-ADT	0.18 0.50 SYN-SYN	IND	SYN	SYN	> 128/64/16
**KP37**	ST395	OXA-48 CTX-M-15 RmtC,QnrB	INCR	0.56 1 ADT-IND	0.37 0.62 SYN-ADT	0.125 0.56 SYN-ADT	IND	SYN	SYN	128/32/16
**KPB6**	ST15	NDM-1 CTX-M-15, TEM. SHV, CIT rmtB-C, QnrS	INCR, INCA/C	0.5 0.75 ADT-IND	0.56 0.75 ADT-ADT	0.26 0.5 SYN-SYN	IND	SYN	SYN	256/32/8

*IND* Indifference, *SYN* Synergistic, *ADT*Adittive. *ERT* Ertapenem, *MEM* Meropenem, *COL* Colistin

According to the checkerboard results; in vitro synergistic activities were observed with ertapenem/meropenem (5/10 isolates), meropenem/colistin (7/10) and ertapenem/meropenem/colistin (9/10); no antagonism was observed for all combinations. For the time-kill assay results; synergism and bactericidal effects were observed with meropenem/colistin and with ertapenem/meropenem/colistin combinations (10/10), and an indifference effect was observed with the ertapenem and meropenem combination (10/10) (Table 2).

**Fig. 1:**
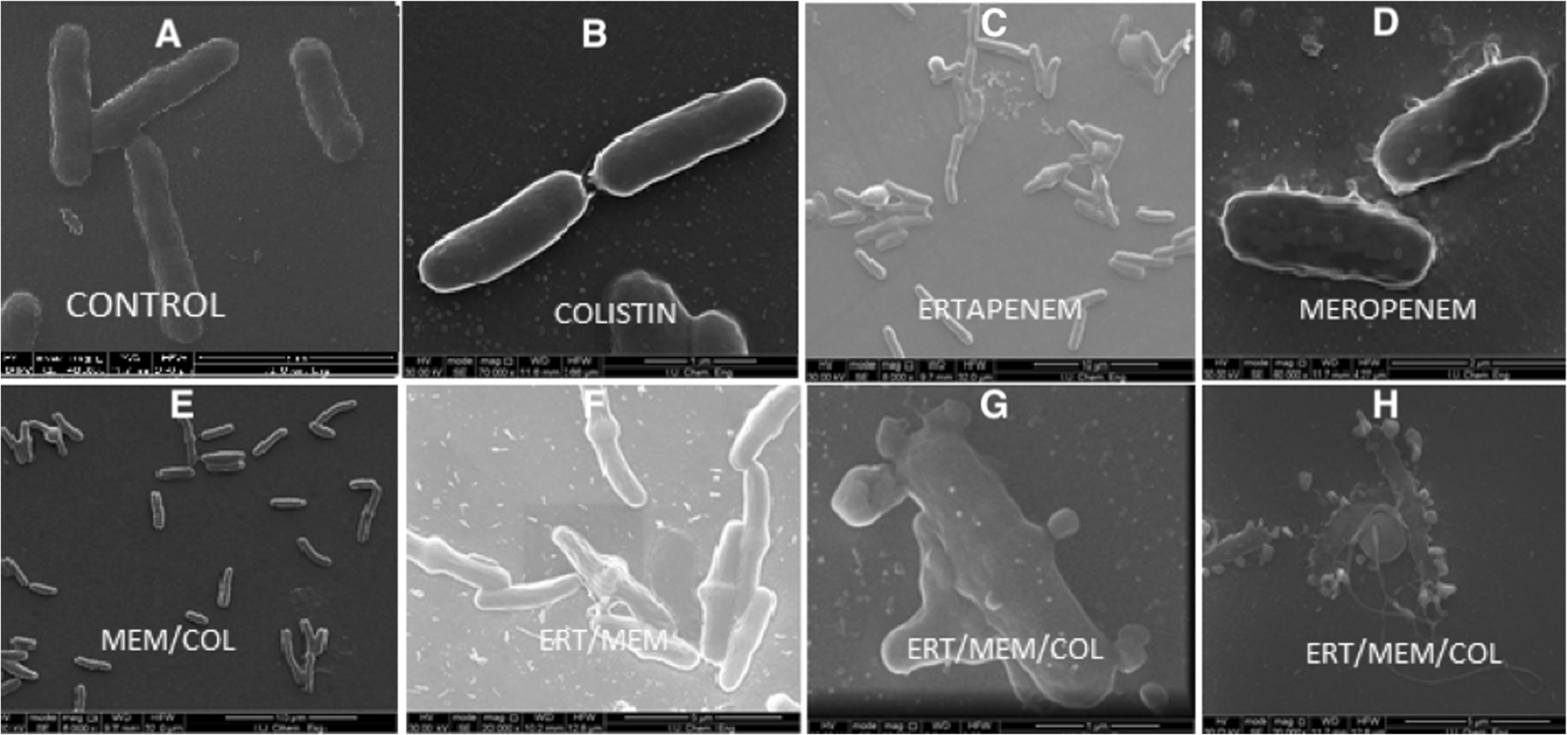
**a**. SEM images of *K. pneumoniae* B6. **b**. Control (absance of drug therapy): Smooth cell surface. **c**. Precense of colistin: Minimal pits and protrusions. on the cell surface. **d**. Precense of ertapenem: Minimal pits and protrusions. **e.** Precense of meropenem: Minimal pits and protrusions **f**. Precense of meropenem and colistin: Minimal pits and protrusions..**g**. Precense of ertapenem, meropenem: Minimal pits and cantral bulginess. **h**. Precense of ertapenem, meropenem and colistin: Severe cell membrane demage and celuler lysis. ERT:Ertapenem, MEM:Meropenem, COL:Colistin

Analysis of *K. pneumoniae* isolate B6 with SEM revealed morphologic changes on the cell surface after treatment with each antibiotic both alone and in combinations and in the absence of antibiotics. The cell surface in the control group was smooth, whereas the cells treated with ertapenem, meropenem, and colistin alone showed uneven surface bulges (Fig. 1). The combination treatment with meropenem/colistin, ertapenem/meropenem and especially ertapenem/meropenem/colistin combinations caused more significant damage to the cell surface, with numerous bulges and roughness and enlargement in the central region of their walls and cellular lysis. The cells had filaments in the central region of their cytoplasms. The filament formations in our results appeared as lightened areas in the mid-region of the bacterial cells. *K. pneumoniae* cells showed the development of numerous breaks in the cell wall, suggesting that the primary target of this treatment was the outer membrane of these Gram-negative bacteria (Fig. 1). Treatment was disrupting the outer membrane and cell wall and this led to the death of the cells [20].

**Fig. 2:**
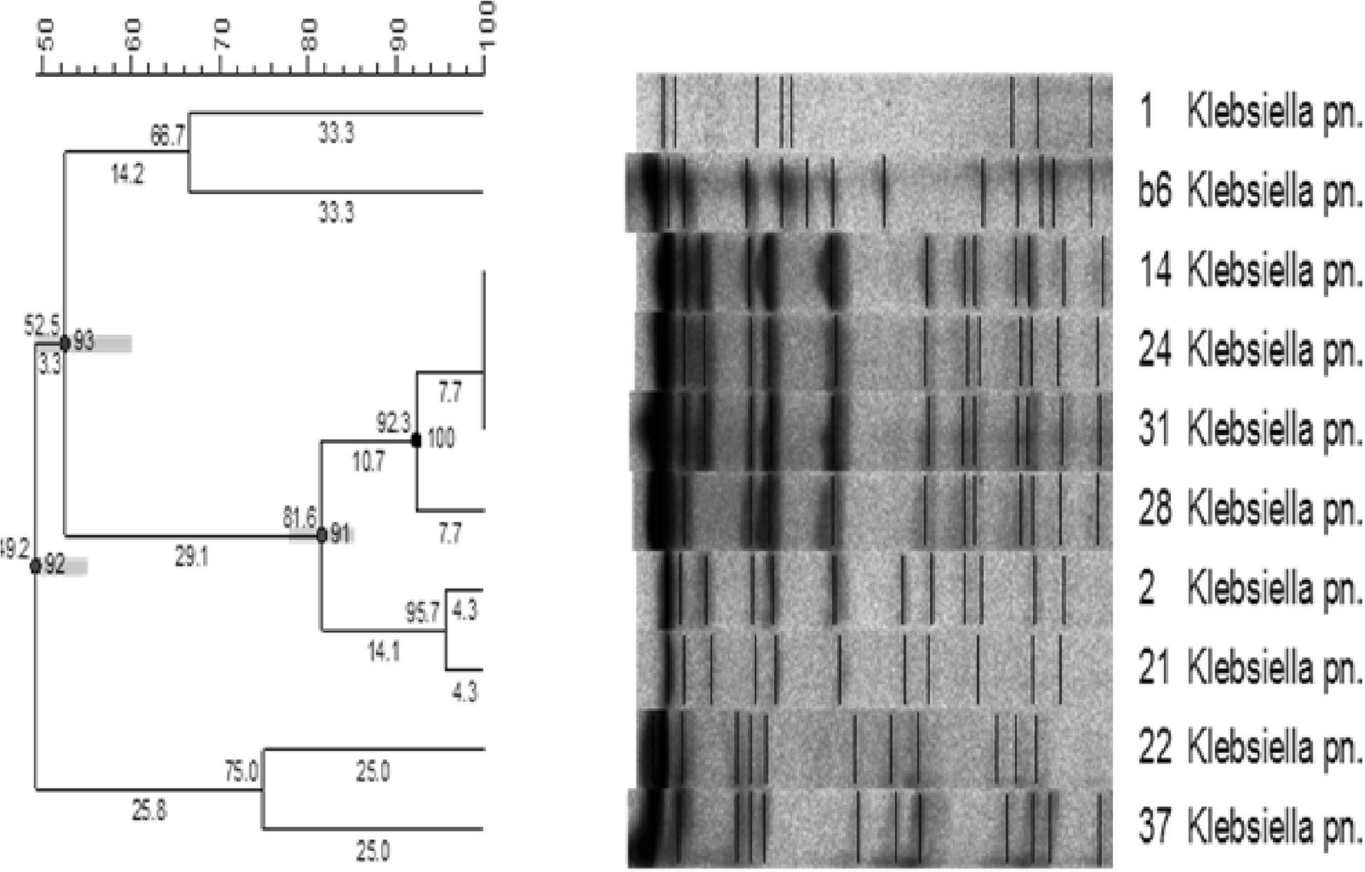
Results of PFGE

PFGE analysis of the 10 isolates revealed the existence of five different genetic clusters (Fig. 2: a-e). Four sequence types were obtained through MLST (ST14(*n* = 1), ST15(*n* = 1), ST101(*n* = 6), ST395(*n* = 2)) (Table 2).

All of the isolates were positive for bla_CTXM-15_, bla_SHV_ beta lactamases. Eight of the isolates also coproduced bla_TEM_ beta lactamase. All isolates produced at least one of the two main types of carbapenemases: bla_OXA-48_ (*n* = 8), and bla_NDM-1_ (*n* = 3), one isolate co-produced two carbapenemases, bla_OXA-48_ and bla_NDM-1_. Isolate 37 was carrying *QnrB*, isolate B6 was carrying *QnrS* and *CIT* genes. For all isolates, at least one aminoglycoside resistance-associated gene was detected (*RmtB* (*n* = 1), *rmtD* (*n* = 5), *rmtC* (*n* = 3)) (Table 2).

Regarding the plasmid types, eight isolates possessed an Inc./rep of type R and L. One isolate that expressed bla_NDM-1_ hosted an Inc./rep of type R, H1B-M, F1B and FII, a second isolate that expressed blaNDM-1 possessed an Inc./rep of type R and A/C (Table 2).

### Whole-genome sequencing of *K. pneumoniae* 1 (KPNB1)

WGS was done for KPNB1 that was found to be non-susceptible to all antibiotics tested, including tigecycline, cephalosporins, penicillins, carbapenems, aztreonam, aminoglycosides, quinolones, colistin, and tetracycline. The complete genome of KPNB1 consists of a circular chromosome 5,533,942 base-pairs in length. It was found 100% identical to *Klebsiella pneumoniae subsp. pneumoniae* HS11286 isolated from human sputum in 2011 in Shanghai, China according to the outcomes of complete genome sequence analysis [30]. Three copies of bla_OXA-181_ and one copy of bla_NDM-1 laCTXM-15_ were proven to be on *ISEcp1*. One IS*Ecp1*-bla_OXA-181_ mobile element had disrupted the *mgrB* regulatory gene, accounting for resistance to colistin, and the strain carried the genes bla_OXA-181_, bla_CTXM-15_, bla_NDM_, *arr-3, aac (6′)-Ib-cr, rmtF* and *catB1*. The complete genome of the plasmid that codes bla_CTXM-14_ is 122,799 bp (49.5% G_C content) in length. The outcomes of the study showed that an blaNDM-type carbapenemase gene was carried on the plasmid. Outcomes of MLST analysis revealed that the strain was an ST14 sequence-type isolate.

## Discussion

Global spread of MDR *K. pneumoniae* is an epidemiologic challenge. Accurate treatment, along with infection control measures all have a role in preventing the development of resistant strains. Reduced mortality is associated with appropriate antimicrobial therapy and with the initiation of this therapy in the early stage of infection [31].

In this study, by the checkerboard tests, synergistic effects were observed for all drug combinations aganist three NDM-producing isolates. Additive effects were observed with ertapenem-meropenem and meropenem-colistin combinations against one bla_NDM_ producer which has CTXM-15, TEM, SHV, CIT, rmtB-C, QnrS resistance genes. While variable effects were observed with double carbapenem, synergistic effects were observed with triple combination against eight bla_OXA-48_ producing *K.pneumoniae* strains. According to time kill studies synergistic effects were observed with both meropenem-colistin and ertapenem-meropenem-colistin combinations against both bla_NDM_ and bla_OXA-48_ producers. A indifference effects were observed with ertapenem-meropenem against both bla_NDM_ and bla_OXA-48_ producing *K.pneumoniae* strains (Table 2).

In this study, regimens of colistin combined with one or two carbapenem exhibited a high level of synergism, even in the presence of colistin resistance. The results were in agreement with other studies that investigated effect of triple (Ertapenem plus meropenem plus colistin) and double combinations (meropenem plus colistin) [32, 33].

In several studies, the use of double carbapenem regimen has been proposed as a valid therapeutic option in the treatment of KPC producing, multi-drug resistant *Klebsiella pneumoniae* clinical isolates [34–36]. On the other hand, in a study, bactericidal activity was reported from 17 to 20% with double combinations of ertapenem, meropenem and imipenem against OXA-48-producing *Klebsiella pneumoniae* clinical isolates. In the same study, no combination axhibited antagonism [37].

In this study, different sequence types with bla_NDM_-positive isolates obtained with MLST and PFGE results indicate different geographic origins and horizontal transfer of resistance elements. Over 50% of NDM-producing *K. pneumoniae* isolates were reported from India belonging to either ST11 or ST147 [38]. ST14, ST101, and ST395 have been reported in European countries. *K. pneumoniae* ST14-coproducing blaNDM-1 was reported in India, the United Kingdom, Sweden, and the United Arab Emirates [39, 40] ST15 *K. pneumoniae* isolates were reported in Bulgaria, Croatia, Czech Republic, Denmark, Hungary, Italy, The Netherlands, and Spain, China, South Korea, Malaysia, Singapore, Thailand, and Vietnam [41]. However, it is noteworthy that, this study was presented at the 26th European Congress of Clinical Microbiology and Infectious Diseases (ECCMID 2016. (EPO234) as the first report of ST 15 NDM-producing *K. pneumoniae* [42, 43].

This study confirmed that acquisition and spread of resistance genes are associated with mobile genetic elements such as plasmids and transposons. The bla_NDM-1_ and bla_OXA-48_ genes are carried on plasmids, and spread by transferable elements between diferent plasmids, and is then further spread in multiple bacteria via plasmids. The results obtained in this study showed that all isolates are expressed Inc. R type plasmid corelated with the previous study which was conducted with carbapenem resistant *Klebsiella pneumoniae* strains [44]. Also, ınc L is the second most common plasmid type (9/10) which was previously shown to be responsible for transfer of bla_OXA-48_ type carbapenemase in a ST395 *K.pneumoniae* strain [44, 45]. One isolate that expressed bla_NDM-1_ and bla_CTX-M-15_ hosted an Inc./rep type of R, H1B-M, F1B and FII. IncFIIK plasmids, which are important vehicles of multiple antibiotic resistance genes, have been shown to be resposible for transferring bla_CTxM-15_ and other resistant genes [46]. Otherwise, molecular epidemiologic studies have reported that IncA/C, IncFIIK, IncL/M, and IncH1 type plasmids are responsible for the horizontal spread of blaNDM-type carbapenemase [47, 48]. Also, the IncX3-type plasmid has been reported to be responsible for horizontal transfer of the bla_NDM_ gene in several studies [49].

In this study, it was shown that genes encoding 16S rRNA methyltransferase were accompanied by the β-lactamase enzymes (10/10). Otherwise, co-existence of the blaNDM-1 and *rmtC* genes was reported in Turkey in 2016 coraleted with this study [50]. However, it has been shown that genes encoding 16S rRNA methyltransferase and β-lactamase enzymes are usually transported by the same plasmid. So this situation allows for considerable effectiveness of aminoglycosides in combination with carbapenems or colistin [51].

The present study is the first Turkish report of blaOXA–181-type carbapenemase causing colistin resistance. blaOXA-181 differs from blaOXA-48 by four amino 80 acid substitutions. Inactivation of *mgrB* has recently been associated with resistance to colistin, and appears to be the most common mechanism for polymyxin resistance in *K. pneumoniae* [52] and it is interesting to note that *mgrB* is disrupted by a functional, IS*Ecp1*-driven blaOXA-181 insertion causing resistance to carbapenems [53, 54]. The emergence of colistin resistance in blaOXA181-producing *K. pneumoniae* has been reported worldwide, which was first reported in 2011 from India [53]. It was reported as the most common carbapenemase followed by blaOXA-48 in Canada between 2011 and 2014 [55–57].

## Conclusion

The ERT/MEM/COL combination was demonstrated to be synergistic and bactericidal. Using double carbapenem antibiotics with colistin further increased the synergistic effect in MDR *Klebsiella pneumonia* strains. This combination might prevent resistance development and secondary effects of colistin monotherapy. Our data could be helpful for clinicians to treat patients with COL-resistant CRKP infections. Our findings suggest that; in vitro synergy tests should be routinely performed in cases of infections due to COL-resistant CRKP strains to select the best antimicrobial combinations. Furthermore, there is a need for long-term multi-centered molecular epidemiologic studies for the recognition of the global spread of antibiotic resistance.

## Data Availability

Not applicable.
